# The effect of rapid high-intensity light-curing on micromechanical properties of bulk-fill and conventional resin composites

**DOI:** 10.1038/s41598-020-67641-y

**Published:** 2020-06-29

**Authors:** Matej Par, Danijela Marovic, Thomas Attin, Zrinka Tarle, Tobias T. Tauböck

**Affiliations:** 1 Department of Conservative and Preventive Dentistry, Center for Dental Medicine, University of Zurich, Plattenstrasse 11, Zurich, Switzerland; 2 Department of Endodontics and Restorative Dentistry, School of Dental Medicine, University of Zagreb, Gunduliceva 5, Zagreb, Croatia

**Keywords:** Composite resin, Dental biomaterials

## Abstract

Rapid high-intensity light-curing of dental resin composites is attractive from a clinical standpoint due to the prospect of time-savings. This study compared the effect of high-intensity (3 s with 3,440 mW/cm^2^) and conventional (10 s with 1,340 mW/cm^2^) light-curing on micromechanical properties of conventional and bulk-fill resin composites, including two composites specifically designed for high-intensity curing. Composite specimens were prepared in clinically realistic layer thicknesses. Microhardness (MH) was measured on the top and bottom surfaces of composite specimens 24 h after light-curing (initial MH), and after subsequent immersion for 24 h in absolute ethanol (ethanol MH). Bottom/top ratio for initial MH was calculated as a measure of depth-dependent curing effectiveness, whereas ethanol/initial MH ratio was calculated as a measure of crosslinking density. High-intensity light-curing showed a complex material-dependent effect on micromechanical properties. Most of the sculptable composites showed no effect of the curing protocol on initial MH, whereas flowable composites showed 11–48% lower initial MH for high-intensity curing. Ethanol/initial MH ratios were improved by high-intensity curing in flowable composites (up to 30%) but diminished in sculptable composites (up to 15%). Due to its mixed effect on MH and crosslinking density in flowable composites, high-intensity curing should be used with caution in clinical work.

## Introduction

The development of materials and techniques in adhesive dentistry follows a continuous trend toward simplification of restorative procedures, as highlighted by the evolution of bulk-fill resin composites^1–3^, universal adhesives^4^, and high-intensity light-curing units^5^. The benefits made possible by these advancements reach beyond mere improvements in the cost-effectiveness of the restorative treatment, as simplified procedures also reduce the risk of iatrogenic errors^6^.

The evolution of light-curing protocols has been following the technological improvements of light-curing devices, which generally involved increasing radiant exitance and narrowing the emission spectrum to the useful wavelength range^7^. The approach of shortening exposure time by increasing radiant exitance has raised justified concerns related to polymerization shrinkage stress, which motivated investigations on various modulated light-curing protocols as a potential means for minimizing shrinkage stress and its detrimental consequences^8^. Although laboratory studies have demonstrated convincing evidence for shrinkage stress reduction attained by using various modulated light-curing protocols^9–11^, their benefits were less clear in the clinical setting^12,13^. Due to the lack of evidence for clinical benefits, modulated light-curing protocols could not become generally accepted, whereas clinical practices are dominated by continuous light-curing protocols with radiant exitances of about 1,000 mW/cm^2^^14^.

In the course of the development of light-curing units, the term “high-intensity” has acquired an ambiguous meaning. During the last two decades, radiant exitances of LED curing units have gradually increased for a whole order of magnitude^14^, leading to the corresponding adjustments to the meaning of “high-intensity” in the literature. In the 1990s, the radiant exitances of 100–200 mW/cm^2^ were common for early LED curing units, leading to light-curing protocols of 450 mW/cm^2^ being regarded as “high-intensity”^15,16^. During the 2000s, the range of radiant exitances associated with the term “high-intensity” shifted to 1,000–2,000 mW/cm^2^^17–20^. As radiant exitances of 1,000–2,000 mW/cm^2^ have nowadays become commonplace, the term “high-intensity” is currently being used to denote values over 2,000 mW/cm^2^^21,22^. The described evolution of terminology refers mainly to LED curing units, which have dominated both the dental market and practice during the last decade. Another type of high-performance curing units, namely plasma-arc curing units, with radiant exitances reaching up to 7,500 mW/cm^2^^11,23,24^ have also been present during that time, but never became widely accepted by dental practitioners.

Changing the parameters of light-curing is related to two main concerns: (I) on curing effectiveness throughout the composite increment^19^, and (II) on possible differences in the crosslinking density of the polymeric network resulting from different radiant exitances^18^. The depth-dependent curing effectiveness is commonly evaluated by comparing microhardness (MH) or degree of conversion between the top and bottom specimen surfaces^25^, whereas crosslinking density is usually indirectly evaluated through ethanol softening, i.e. the MH decrease caused by immersion in ethanol^26^. Whereas the effect of modulated light-curing protocols on MH and crosslinking density of resin composites has been studied extensively^27–32^, the effect of high-intensity continuous curing (above 3,000 mW/cm^2^) on MH and crosslinking density of contemporary bulk-fill composites has not been investigated up to date.

The aim of this study was to compare the effect of a high-intensity and a conventional curing protocol on MH values measured 24 h post-cure, bottom/top MH ratio, and MH decrease due to ethanol softening for conventional and bulk-fill composites, including two bulk-fill composites specifically designed for high-intensity (sometimes regarded as “ultra-fast”^5,33^) light-curing. The null hypotheses assumed that the aforementioned properties would not be affected by (I) curing protocol, and (II) composite material.

## Materials and methods

### Composite materials and light-curing protocols

**Figure 1:**
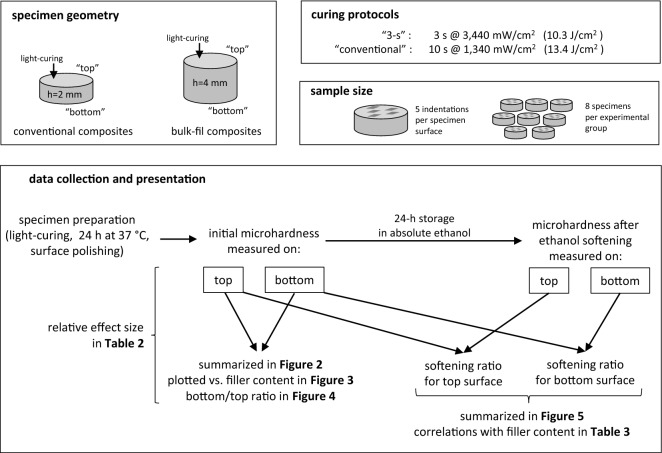
Schematic representation of the study design.

Seven resin composites were investigated in order to include materials representative for different material classes, i.e. conventional, bulk-fill, sculptable, and flowable composites (Table 1). Two of the investigated materials (Tetric PowerFill and Tetric PowerFlow) are specifically designed for high-intensity light-curing. The schematic overview of the study design is shown in Fig. 1.

**Table 1:** Resin composites investigated in this study.

Composite viscosity	Composite type	Composite name (abbreviation)	Filler content (wt%/vol%)	Resin matrix	Photoinitiator	Manufacturer	Shade/LOT No.
Flowable	Conventional	Tetric EvoFlow (TEF)	58/31	Bis-GMA, UDMA, decandioldimethacrylate	CQ/amine	Ivoclar Vivadent, Schaan, Liechtenstein	A2/Y15650
	Bulk-fill	x-tra base (XB)	75/60	Bis-EMA, UDMA	CQ/amine	Voco, Cuxhaven, Germany,	Universal/1,932,130
		Tetric PowerFlow (PFW)	68/46	Bis-GMA, Bis-EMA, UDMA	CQ/amine + Ivocerin	Ivoclar Vivadent, Schaan, Liechtenstein	IVA/Y15023
Sculptable	Conventional	Ceram.x (CER)	76/57	Methacrylate modified polysiloxane, dimethacrylate resin	CQ/amine	Dentsply Sirona, Konstanz, Germany	A2/0,189
	Bulk-fill	Filtek One Bulk Fill (FIL)	77/59	UDMA, aromatic UDMA, DDDMA, proprietary AFM	CQ/amine	3 M Espe, St. Paul, MN, USA	A2/NA60719
		Tetric EvoCeram Bulk Fill (TECBF)	77/54	Bis-GMA, Bis-EMA, UDMA	CQ/amine + Ivocerin + Lucirin TPO	Ivoclar Vivadent, Schaan, Liechtenstein	IVA/Y16932
		Tetric PowerFill (PFL)	77/54	Bis-GMA, Bis-EMA, UDMA, propoxylated bisphenol A dimethacrylate, DCP, β-allyl sulfone AFCT agent	CQ/amine + Ivocerin + Lucirin TPO	Ivoclar Vivadent, Schaan, Liechtenstein	IVA/X56571

*Bis-GMA* bisphenol-A-glycidyldimethacrylate, *UDMA* urethane dimethacrylate, *Bis-EMA* ethoxylated bisphenol-A-dimethacrylate, *DDDMA* 1, 12-dodecanediol dimethacrylate, *AFM* addition fragmentation monomer, *DCP* tricyclodecane-dimethanol dimethacrylate, *AFCT* addition-fragmentation chain transfer, *CQ* camphorquinone, *TPO* 2,4,6-trimethylbenzoyldiphenylphosphine oxide.

Two light-curing protocols were investigated: the protocol designated as “3-s” involved light-curing for 3 s with a radiant exitance of 3,440 mW/cm^2^ (radiant exposure = 10.3 J/cm^2^), whereas the protocol designated as “conventional” involved light-curing for 10 s with a radiant exitance of 1,340 mW/cm^2^ (radiant exposure = 13.4 J/cm^2^). Light-curing was performed using a violet-blue LED curing unit (Bluephase PowerCure, Ivoclar Vivadent, Schaan, Liechtenstein; emission wavelength range: 390–500 nm). The radiant exitance values were measured and periodically verified using a calibrated and NIST-referenced UV–Vis spectrophotometer system (MARC; BlueLight Analytics, Halifax, Canada).

### Composite specimen preparation

Cylindrical composite specimens (diameter = 6 mm, height = 2 or 4 mm for conventional and bulk-fill composites, respectively) were prepared by casting uncured composites in custom-made polyoxymethylene (POM) moulds, covering the mould openings with Mylar foil, and flattening the specimen surfaces using glass plates^34^. The composite cylinders were irradiated from one side according to the curing protocols described above. The irradiated side was denoted as “top”, whereas the opposite side was denoted as “bottom”. The distal tip of the curing unit light guide was positioned directly above the specimen; therefore, radiant exitance from the curing unit can be considered to be equal to irradiance received by the top specimen surface. The composite specimens were stored dry in the dark at 37 °C for 24 h, in order to complete the post-cure reaction^35^. Subsequently, both top and bottom specimen surfaces were wet-ground for 3 min using P4000 silicon carbide (SiC) paper at low speed (30 rpm) to avoid artificially increasing the extent of polymerization due to heating^36^. After grinding, the top and bottom specimen surfaces were polished using an 0.05-micron aluminium oxide suspension (MasterPrep; Buehler, Lake Bluff, IL, USA) and polishing cloth (MasterTex; Buehler) for 3 min at 60 rpm. Eight composite specimens per experimental group were prepared (n = 8).

### Microhardness measurements

Knoop MH was measured after the above-described preparation procedure and after individual specimen storage for 24 h in 5 mL of absolute ethanol (Merck, Darmstadt, Germany) in the dark at 37 °C. Measurements were performed on the top and bottom specimen surfaces using a digital hardness tester (model no. 1600–6106; Buehler). Indentations were made under a load of 100 g and a dwell time of 20 s at random positions around the centre of the specimen. The indentations were evaluated within 2 min after preparation, with a resolution of 0.015 µm. Per each specimen surface, five replicate indentations were performed and their mean values were treated as a statistical unit. This approach was used to minimize the effect of the heterogeneity of MH and crosslinking density across the specimen surface^37^. The bottom/top ratio for initial MH values was calculated as a measure of depth-dependent curing effectiveness^38^. The ratio between MH measured after ethanol softening and initial MH was calculated as a measure of crosslinking density^39^.

### Statistical analysis

Normality of distribution and homogeneity of variances were checked using Levene’s and Shapiro–Wilk’s tests, respectively. A two-way ANOVA with partial eta-squared statistics was performed to evaluate the effect of the factors “material” and “curing protocol” on the following outcome variables: initial MH, bottom/top MH ratio, and ethanol/initial MH ratio. The mean values of the aforementioned outcome variables were compared among the combinations of factors “material” and “curing protocol” using one-way ANOVA with Tukey’s adjustment for multiple comparisons. Pearson’s correlation analysis was used to investigate the relationship between the composite’s filler content as a predictor variable, and initial MH and ethanol/initial MH ratios as outcome variables. The statistical analysis was performed using SPSS (version 20, IBM, Armonk, NY, USA) at an overall level of significance of α = 0.05.

## Results

Partial eta-squared values, as a measure of the relative effect size for the factors “material” and “curing protocol” on initial MH, bottom/top MH ratio, and ethanol/initial MH ratio are shown in Table 2. Both factors, as well as their interactions, had a significant effect on all of the outcome variables. The factor “material” showed a higher effect size compared to “curing protocol” for all outcome variables. For the outcome variables that were analysed separately for the top and the bottom specimen surface, i.e. initial MH and ethanol/initial MH ratio, a higher effect size of the factor “curing protocol” was identified for the bottom compared to the top specimen surface.

**Table 2:** Partial eta-squared values describing relative effect size of factors “material”, “curing protocol”, and their interactions on initial microhardness, bottom/top ratio of initial microhardness, and ethanol/initial microhardness ratio.

	Initial microhardness				Bottom/top ratio for initial microhardness		Ethanol/initial microhardness ratio			
	Top surface		Bottom surface				Top surface		Bottom surface	
	p	Partial η^2^	p	Partial η^2^	p	Partial η^2^	p	Partial η^2^	p	Partial η^2^
Factor										
Material	< 0.001	0.981	< 0.001	0.971	< 0.001	0.952	< 0.001	0.982	< 0.001	0.982
Curing protocol	< 0.001	0.648	< 0.001	0.718	< 0.001	0.767	< 0.001	0.285	< 0.001	0.403
Material × curing protocol	< 0.001	0.705	< 0.001	0.599	< 0.001	0.817	< 0.001	0.801	< 0.001	0.899

**Figure 2:**
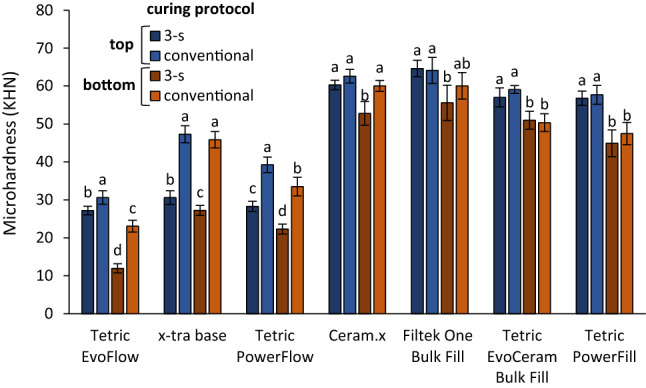
Initial microhardness (mean values ± standard deviation). Same letters denote statistically homogeneous groups within each material.

Initial MH values measured on the top and bottom specimen surfaces are shown in Fig. 2. The MH values (KHN) ranged between 12.0–47.3 for the flowable composites, and 44.9–64.6 for the sculptable composites. In the group of flowable composites, the 3-s curing resulted in 11–35% lower top MH values and 33–48% lower bottom MH values compared to the conventional curing. On the other hand, in the group of sculptable composites, a significant effect of the curing protocol was identified only for the MH values measured on the bottom specimen surface of CER, for which the 3-s curing resulted in 12% lower MH compared to the conventional curing. The remaining three sculptable composites (FIL, TECBF, and PFL) showed no effect of the curing protocol on their MH values.

**Figure 3:**
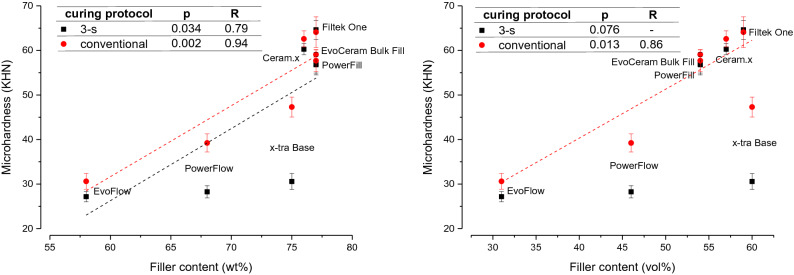
Plots of initial microhardness measured on top specimen surface vs. filler content (left: weight percentage; right: volume percentage) and results of Pearson correlation analysis. Error bars represent ± 1 standard deviation. Dashed lines represent statistically significant correlation functions.

In Fig. 3, initial MH values measured on the top specimen surface are plotted as a function of filler content. Significant positive correlations were identified between MH values and filler weight percentage, with Pearson’s correlation coefficients of 0.79 and 0.94 for the 3-s and the conventional curing, respectively. A comparatively weaker association was identified between MH and filler volume percentage, as a significant correlation was obtained only for the conventional curing, with a correlation coefficient of 0.86.

**Figure 4:**
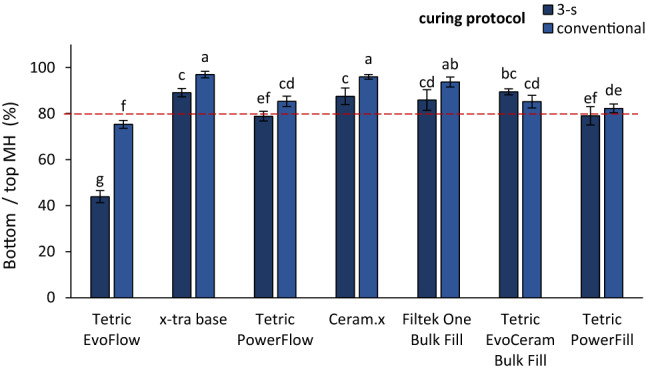
Bottom/top ratios for initial microhardness (mean values ± standard deviation). Same letters denote statistically homogeneous groups. Dashed red line denotes the 80% bottom/top MH threshold.

Figure 4 shows bottom/top ratios for initial MH values. Except for TEF, all of the investigated composites reached or surpassed the 80% bottom/top MH threshold. The bottom/top MH ratios ranged between 43.9–89.4% for the 3-s curing and 75.3–96.9% for the conventional curing. TECBF and PFL showed no significant effect of the curing protocol on the bottom/top MH ratio, whereas TEF, XB, PFW, CER, and FIL showed significantly lower bottom/top MH ratios for the 3-s compared to the conventional curing.

**Figure 5:**
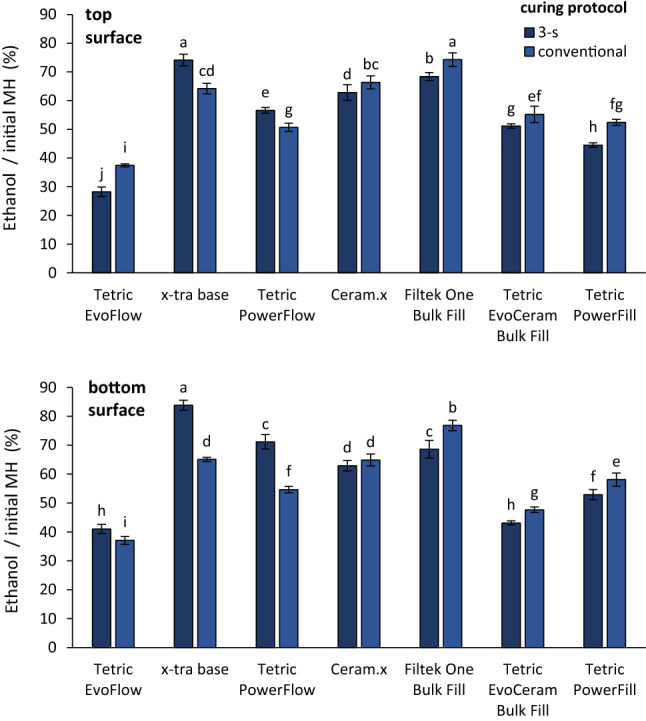
Ratio of microhardness measured after ethanol immersion and initial microhardness (mean values ± standard deviation). Same letters denote statistically homogeneous groups within a specimen surface.

Figure 5 shows the ratios of MH values measured after ethanol immersion and initial MH values, separately for the top and bottom specimen surfaces. On top specimen surfaces, the MH values after ethanol softening ranged between 28.2–74.2% for the 3-s curing and 37.4–74.3% for the conventional curing. On bottom specimen surfaces, ethanol/initial MH ratios ranged between 41.0–83.8% for the 3-s curing and 37.1–76.8% for the conventional curing. In the group of flowable composites, the 3-s curing produced significantly higher ethanol/initial MH ratios compared to the conventional curing, except for TEF at the top specimen surface. In the group of sculptable composites, the 3-s curing produced significantly lower ethanol/initial MH ratios compared to the conventional curing, except for CER at the bottom specimen surface.

Table 3 shows the results of Pearson’s analysis for the correlation of the composite’s filler content and ethanol/initial MH ratios. Significant positive correlations were identified mostly for the conventional curing protocol, whereas the 3-s curing protocol yielded a significant correlation only for ethanol/initial MH ratios measured on the top specimen surface and filler load expressed in vol%.

**Table 3:** Pearson’s R values for correlations of filler content and ethanol/initial microhardness ratio (p-values in brackets).

Specimen surface	Ethanol/initial microhardness ratio			
	Top		Bottom	
	3-s curing	Conventional curing	3-s curing	Conventional curing
**Filler content**				
Weight %	N. S	0.79 (0.036)	N. S	N. S
Volume %	0.85 (0.015)	0.89 (0.007)	N. S	0.84 (0.019)

*N.S.* not significant.

## Discussion

This study investigated the effect of a high-intensity (“3-s”) and a conventional curing protocol on micromechanical properties (MH), depth-dependent curing effectiveness (bottom/top MH ratio), and crosslinking density (ethanol/initial MH ratio) of bulk-fill and conventional composites. As the evaluated properties were significantly affected by different curing protocols and composite materials, both null hypotheses were rejected.

The analysis of relative influences of the factors “material” and “curing protocol” shows that the factor “material” was more influential for all of the outcome variables (initial MH, bottom/top MH ratio, and ethanol/initial MH ratio), whereas significant interactions between the factors “material” and “curing protocol” show that the effect of the curing protocol was inconsistent among materials. These results indicate that differences in material composition were a more important source of variability in mechanical properties^40,41^ than the curing protocol, and that the effect of changing curing parameters was material-dependent^42^. It should be noted that due to the attenuation of curing light on its way through the composite specimen^43,44^, the effect size of the curing protocol was higher on the bottom specimen surface compared to the top surface. This indicates that the influence of curing parameters on MH and ethanol softening can be expected to increase as the distance from the irradiated surface is increased. The potential issues arising from uneven distribution of curing light across the light guide tip were avoided by using a curing unit that features a built-in light homogenizer. The homogeneous distribution of light intensity and wavelengths has been described in the manufacturer’s publications and additionally confirmed in a preliminary test in which the same light profiles were obtained on randomly selected parts of the light guide tip.

Since the material composition was identified as the most influential factor for all outcome variables, Pearson’s correlation analysis was performed in order to explore the relationship between the top surface MH values and the material characteristic that is expectedly the primary determinant for mechanical properties, i.e. filler content^39,41^. High correlations for both weight and volume percentages (R = 0.94 and 0.86, respectively) were identified for the conventional curing. However, the 3-s curing yielded a comparatively lower R-value (0.79) for filler weight percentage, and a non-significant correlation result for filler volume percentage. The weaker correlation for the 3-s curing reflects the fact that the variability introduced by the material-dependent effect of high-intensity curing on the polymer network structure diminished the relative influence of the filler content for determining the composite’s MH. This was especially pronounced in two flowable composites (PFW and XB), for which the scatterplots of initial MH vs. filler content indicate the highest deviations from the correlation line, as well as the highest MH differences between the curing protocols.

The flowable composites demonstrated a notably different effect of the high-intensity curing on the initial MH values compared to the sculptable composites. Whereas only one out of four sculptable composites showed a modest reduction in MH resulting from the 3-s curing compared to the conventional curing (CER on the bottom surface, 12% reduction), a more extensive MH reduction (11–48%) was observed for all of the investigated flowable composites, regardless of the specimen surface. These results can be explained by a higher susceptibility of flowable composites to bimolecular termination that occurs when a large number of free radicals are present simultaneously due to a high initiation rate^45^. Differences in the mobility of reactive species resulting from different viscosities of reaction medium have been considered responsible for the commonly reported finding that polymerization effectiveness tends to be more diminished by the high-intensity light-curing in flowable than in sculptable composites^46,47^. The effects of this phenomenon were identified in all flowable composites investigated in our study, including the composite specifically designed for high-intensity light curing (PFW), which had its MH values diminished for 28–33% when cured using the 3-s compared to the conventional protocol.

Besides the above-described effect of bimolecular termination, the lower MH in the flowable composites resulting from the 3-s compared to the conventional curing could have been caused by its correspondingly lower radiant exposure (10.3 vs. 13.4 J/cm^2^). These values correspond to the clinically used radiant exposures resulting from the pre-defined settings of the curing unit. However, considering the fact that most of the sculptable composites showed statistically similar MH values on both specimen surfaces regardless of the curing protocol, and a notable contrast in behaviour between the sculptable and flowable composites, a higher rate of bimolecular termination in the latter group appears a more likely explanation for the lower MH values produced by the 3-s curing.

On the other hand, lower radiant exposure of the 3-s curing protocol could be the probable cause for significantly lower bottom/top MH ratios attained by the 3-s curing compared to the conventional curing in all composites except TECBF and PFL. According to the commonly accepted criterion of bottom/top MH ratios above 80% indicating acceptable polymerization throughout a composite layer^19,38^, suboptimal curing effectiveness was identified only for TEF, with bottom/top MH ratios of 44% for the 3-s curing and 75% for the conventional curing. Except TEF, all of the investigated composites showed sufficient curing effectiveness regardless of the curing protocol. However, these results of a sufficient cure throughout the manufacturer recommended layer thickness must be considered together with the fact that MH values measured on both the top and bottom specimen surfaces of flowable composites were significantly lower for the 3-s curing compared to the conventional curing. If observed outside the context of absolute MH values, the favourable bottom/top MH ratios may give an overly positive impression about the performance of the flowable composites cured with the 3-s protocol, as this parameter does not capture the considerable negative effect of high-intensity curing on absolute MH values.

In addition to the effect of high-intensity curing on initial MH values being dependent on composite viscosity, the effect of high-intensity curing on ethanol softening also differed between the flowable and sculptable composites. Compared to the conventional curing, the ethanol/initial MH ratio was improved by the 3-s curing for the flowable composites but diminished for the sculptable composites. This pattern of composite viscosity-dependent response to high-intensity curing appears dominant within both composite groups, despite two exceptions observed for the top surface of TEF (change in the opposite direction) and the bottom surface of CER specimens (no effect). Additionally, the influence of the curing protocol on ethanol/initial MH ratios was stronger for the flowable composites compared to the sculptable composites, with relative changes due the 3-s curing compared to the conventional curing amounting to (− 25)–(+ 30)% in the former group, and (− 5)–(+ 15)% in the latter group.

The finding that the flowable composites showed better resistance to ethanol softening when cured with the 3-s compared to the conventional protocol indicates that the polymeric network formed under the conditions of high-intensity curing had improved crosslinking density^48^, despite having diminished MH. Therefore, crosslinking density and absolute MH values were not only affected independently by changing curing parameters but also in opposite directions. A similar observation that increasing the light-curing intensity leads to a diminished extent of polymerization but improved resistance to ethanol softening has been reported in a study in which a model unfilled resin (bisphenol-A-glycidyl dimethacrylate (Bis-GMA) and triethylene glycol dimethacrylate (TEGDMA) in a molar ratio of 1:1) was cured using radiant exitances in a range of 150–600 mW/cm^2^^28^. Such a phenomenon can be attributed to a higher intensity of light-curing leading to a better crosslinking density which in turn impaired the mobility of reactive species^48^, and together with the higher rate of bimolecular termination^45^ diminished the final extent of polymerization. In contrast, higher viscosity of the sculptable composites apparently precluded this potential of high-intensity curing for improving crosslinking density and at the same time diminishing the extent of polymerization.

Whereas the effect of varying intensities of continuous light-curing protocols on ethanol softening has been previously demonstrated for a model unfilled resin^28^, such an effect has not been reported for filled resin composites up to date. This is partly due to scarcity of studies on crosslinking density that investigated continuous curing protocols of varying intensities, as most of the studies on crosslinking density have been focused on comparisons of continuous against modulated curing protocols^18,26–32,49,50^. In the available literature, the effect of light intensity in continuous curing protocols on ethanol softening has been investigated for only two sculptable composites^29,31^, and the results showed no significant effect of variations in radiant exitance between 150–935 mW/cm^2^^29^ and 600–1,200 mW/cm^2^^31^. In both of these studies, significant differences in ethanol softening were identified between continuous and modulated curing protocols, indicating that more radical modifications of curing protocols enabled revealing their effects on ethanol softening. It is, therefore, possible that radiant exitances of continuous curing protocols investigated in these studies were too low to produce a detectable effect on ethanol softening.

The wide range of ethanol/initial MH ratios obtained in this study is in agreement with a previous study on mechanical properties of 11 bulk-fill and conventional composites, which reported ethanol/initial MH ratios amounting to 19–90%^39^. In that study, most of the flowable bulk-fill composites showed significantly lower ethanol/initial MH ratios compared to the sculptable composites. However, a highly-filled conventional flowable composite in that study showed ethanol/initial MH ratios similar to those of sculptable bulk-fill composites, suggesting that filler content was a more important parameter for determining the resistance to ethanol softening than macroscopic material viscosity. In the present study, there was no clear distinction between the flowable and sculptable composites regarding their filler content (XB had a filler load comparable to sculptable composites). Consequently, there was no consistent pattern of ethanol/initial MH ratios being lower for flowable compared to sculptable composites. However, a significant correlation was found between filler content and ethanol/initial MH ratio. This correlation was better identifiable for filler content expressed as volume percentage than weight percentage, as the volume occupied by filler particles is a more direct indicator of the amount of polymer matrix available for ethanol softening. Additionally, the mentioned correlation was better identifiable for the conventional than for the 3-s curing, indicating that ethanol softening of polymeric networks produced by the conventional curing is more directly governed by filler content, whereas in case of the 3-s curing the correlation between ethanol softening and filler content was weakened by the material-dependent response to high-intensity curing.

The effect of the curing protocol on all of the investigated properties (initial MH, bottom/top MH ratio, and ethanol/initial MH ratio) varied among different composites. The observed material-dependent influence of the curing protocol is in line with numerous literature reports on the effect of light-curing parameters affecting micromechanical properties of resin composites to various extents^18,27–30,32^. However, there are also studies that did not identify the effect of light-curing parameters on micromechanical properties despite employing considerably different curing protocols. For example, a study on MH and ethanol softening of commercial composites reported no differences between continuous and soft-start curing^49^, whereas another study reported no effect of a wide range of continuous radiant exitances (0.05–700 mW/cm^2^) produced by UV–visible light sources (320–470 nm) in dynamic mechanical analysis of unfilled Bis-GMA/TEGDMA model resins^51^. Additionally, in a series of studies that investigated the same unfilled model resins and curing protocols, the effect of continuous light-curing (150–600 mW/cm^2^) on crosslinking density was identified by ethanol softening^28^ but could not be detected by the evaluation of glass transition temperature^30^. Such considerations highlight the fact that the outcomes of any investigation of the effect of curing parameters on micromechanical properties are highly dependent on the choice of materials and testing procedures^26,50^, and thus conclusions drawn for specific materials investigated under specific conditions cannot be generalized.

Two of the composites investigated in this study (PFW and PFL) were specifically designed for use with the 3-s curing protocol. The investigated micromechanical properties for these composites were mostly within the ranges obtained for other investigated composites of the corresponding viscosity (flowable for PFW and sculptable for PFL). Although PFL showed ethanol/initial MH ratios at the low-end of the values measured for sculptable composites, its results were within the range obtained for TECBF. The basic components (resin, filler, and photoinitiator system) of PFL and TECBF are very similar, whereas PFL additionally contains a β-allyl sulfone addition-fragmentation chain transfer (AFCT) agent^5^. Therefore, the low ethanol/initial MH ratios identified in PFL are more likely to result from its specific resin/filler/photoinitiator composition than from the effect of an AFCT agent on the formation of the polymer network. An AFCT agent with a different chemistry but similar function of reducing polymerization shrinkage stress is used in FIL^52^. As FIL showed the results in the range of other sculptable composites, it appears that the AFCT agent did not affect micromechanical properties investigated in this study.

As the ethanol softening in this study occurred under much more aggressive conditions compared to the regular aging of composite restorations in the oral environment, the resulting MH changes do not represent the clinical course of material degradation. Instead, the accelerated softening was used as a standard method for indirect evaluation of relative differences in crosslinking density attained under different curing conditions^18,27–32^.

## Conclusions

The effect of high-intensity light-curing on micromechanical properties was markedly dependent on material composition. In flowable composites, high-intensity curing diminished microhardness (for up to 48%) while simultaneously improving crosslinking density (for up to 30%). In contrast, microhardness of sculptable composites was practically unaffected, whereas crosslinking density was moderately reduced. Due to its complex material-dependent effect on micromechanical composite properties, high-intensity curing should be used with caution, especially for flowable composites.

## Data Availability

The datasets generated during and/or analysed during the current study are available from the corresponding author on reasonable request.
